# The Role of Partial Nephrectomy without Arterial Embolization in Giant Renal Angiomyolipoma

**DOI:** 10.1155/2012/365762

**Published:** 2012-03-27

**Authors:** Enis Rauf Coskuner, Burak Ozkan, Veli Yalcin

**Affiliations:** ^1^Department of Urology, Acibadem University School of Medicine, Acibadem Bakirkoy Hospital, Halit Ziya Usakligil Cad. No: 1, Bakirkoy, 34140 Istanbul, Turkey; ^2^Department of Urology, Acibadem Bakirkoy Hospital, Halit Ziya Usakligil Cad. No: 1, Bakirkoy, 34140 Istanbul, Turkey

## Abstract

Angiomyolipoma is a benign neoplasm composed of varying admixtures of blood vessels, smooth muscle cells, and adipose tissue. Because of an increased risk of spontaneous haemorrhage, surgical approach is needed greater than 4–8 cm size. We here report our partial nephrectomy experience in the 24 cm size giant angiomyolipoma. 26-year-old woman referred to our clinic with a 24 cm size angiomyolipoma in her lower pole of right kidney. The inferior vena cava was deviated to the left by the mass. All the blood tests were normal and we offered her the choices of partial nephrectomy or nephrectomy. Right subcostal approach was used. The patient underwent resection of the mass with a safety region of 1 cm. Frozen section evaluation was consistent with angiomyolipoma and free for surgical margin. Warm ischemia time was 35 min. and intraoperative bleeding volume was 200 cc. Postoperative 2nd day the drain was taken and hospital stay was 4 days. In literature we observed very rare angiomyolipoma cases with such a large dimension treated by partial nephrectomy without arterial embolization. If technically suitable partial nephrectomy is the main chioce in this kind of benign lesions in young patients.

## 1. Introduction

Angiomyolipoma (AML) is a rare benign neoplasm, typically composed of varying admixtures of blood vessels, smooth muscle cells, and adipose tissue (1). The average size of these tumors is 2 to 8 cm, with cases involving tumors of up to 20 cm reported in literature [1, 2].

Because of an increased risk of spontaneous haemorrhage, selective transcatheter arterial embolization (TAE) or surgical approach are needed greater than 4 to 8 cm size. We here report our partial nephrectomy experience in the 24 cm size giant angiomyolipoma.

## 2. Case Report

**Figure 1:**
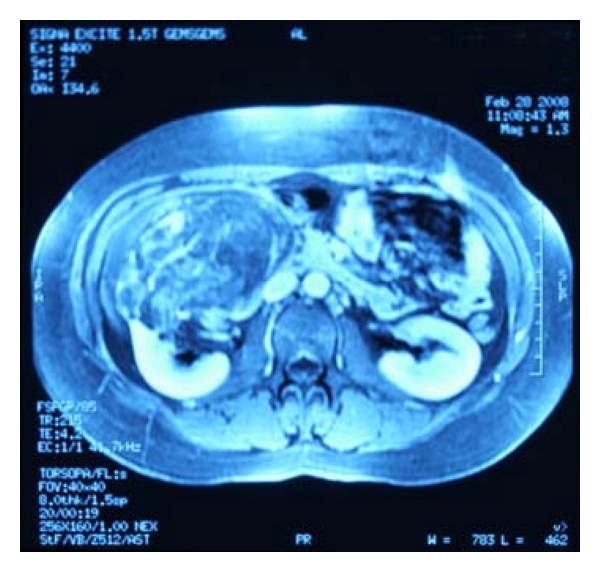
MRI showing a right retroperitoneal mass.

**Figure 2:**
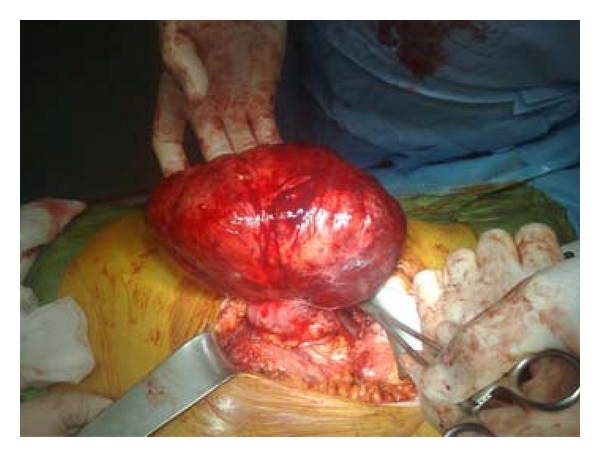
Intraoperative view of the AML.

A 26-year-old woman without the tuberous sclerosis complex (TSC) presented with abdominal fullness, right upper quadrant pain and constipation. On physical examination, a right upper quadrant mass was palpated bimanually. Computerized tomography of abdomen demonstrated a 24 cm size angiomyolipoma that was arising from the lower pole of the right kidney (Figure 1). The inferior vena cava was deviated to the left by the mass. All the blood tests were normal and we offered her the choices of partial nephrectomy or nephrectomy. Right subcostal approach was used. After peritoneal incision the mesocolon was reflected medially along the line of Toldt. Clear uninvolved plane was found between the mass and the vena cava and dissected. The renal artery and vein were occluded with a bulldog clamp. The patient underwent resection of the mass with a safety region of 1 cm. Frozen section evaluation was consistent with angiomyolipoma and free of surgical margin. The collecting system and renal defect were closed (Figure 2).

**Figure 3:**
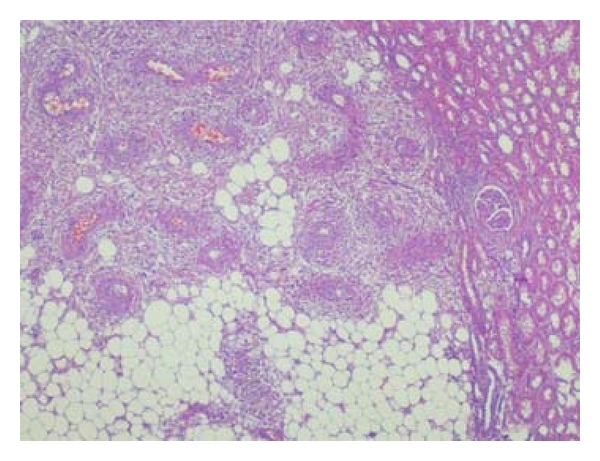
Microscopic view of the mass.

Histopathology showed an encapsulated soft tissue mass which was measured 24 cm at its greatest dimension and weighed 3425 g. The lesion was characterized microscopically by a proliferation of cells, including those demonstrating adipose and smooth muscle differentiation, with the formation of thick-walled vascular structures (Figure 3).

**Figure 4:**
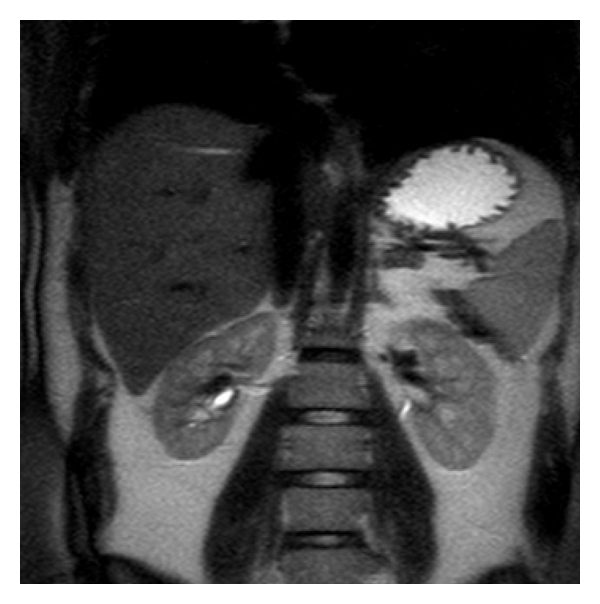
MRI of the right kidney, 6 months after the operation.

Warm ischemia time was 35 min and intraoperative bleeding volume was 200 cc. The patient's postoperative course was uneventful. Postoperative 2nd day the drain was taken and she was discharged home on postoperative day 4.6 months later we evaluated the right kidney as a normal one except postoperative differences (Figure 4).

## 3. Discussion

Renal AML is a benign mesenchymal tumor, identified in less than 0.2% of the general population and a prevalence of 0.3% to 3% of all surgical resected renal tumors [1]. Twenty percent of these tumors are seen in patients with TSC and majority of patients with TSC (80%) develop AMLs [3]. Some authors have recommended intensive intervention for patients with these tumors and others have reported that all of these tumors do not necessarily require intervention [4, 5].

In general asymptomatic AMLs with a diameter of 4 cm or smaller can be followed by imaging studies. Active surveillance can be performed even for large AMLs which do not uniformly become symptomatic until reaching more than 8 cm [6]. For larger tumors, particularly if the patient is symptomatic, surgical intervention should be considered. A nephron-sparing approach, via partial nephrectomy or TAE, is preferred in patients with small AMLs requiring intervention because of symptoms, in patients with tuberous sclerosis or multicentric AMLs, and in patients whom preservation of renal function is important.

TAE is a safe and tolerable procedure it can be done to decrease the risk of bleeding without loss of renal function. After TAE, the recuurence is infrequent but additional treatment may be necessary [7, 8]. The risk of unexpected large ischemic changes should be kept in mind [6]. Lifelong surveillance for recurrance is essential after AML embolization [7, 8].

Recently several cases have been reported for whom nephron-sparing surgery was performed. Especially in sporadic renal AML, this technique offers preservation of renal function with acceptable complication and recurrence rates. Preoperative embolization of the large tumors is recommended to avoid excess blood loss during surgery [9]. But the reduction in tumour volume is only modest (28%) [10].

In our case report we performed a partial nephrectomy procedure in a young female patient without tuberous sclerosis complex. We met very rare AML cases with such big dimensions that were treated by partial nephrectomy without arterial embolization in our literature survey.

Patient's age, strong diabetes history in her family, the anatomical localisation of the tumor in the kidney with the adjacent organs were the main reasons for the decision of the nephron-sparing surgery. We did not try preoperative embolization because of its modest effect on tumor size and to avoid additional burden for our patient. Although our intraoperative bleeding volume was acceptable, we believe that preoperative embolization may reduce this risk.

The management of AML is a complex procedure especially in large or multiple ones. TAE is effective at controlling acute haemorhage but has limited value in the long term management. It may help us before surgical intervention to prevent hemorrhage. New treatment modalities such as focused ablation and the use of antiangiogenic molecules should be further explored. Although life-threatening hemorrhage or fistulas of the collecting system are the main complications partial nephrectomy may be the best choice if the patient's anatomy is available and your surgical experience is enough about nephron-sparing surgery.
